# Association of exposure duration, modality, and polycythaemia with hyperuricaemia among han male immigrants at high altitudes: a cross-sectional study

**DOI:** 10.1080/07853890.2025.2543065

**Published:** 2025-08-05

**Authors:** Yanlin Zhu, Liwen Mo, Jie He, Xianglian Li, Yi Li, Dan Xiao, Huichang Jia, Jian Li, Fan Zhang, Yue Cheng

**Affiliations:** Department of Nephrology, General Hospital of Western Theater Command, PLA, Chengdu, China

**Keywords:** Qinghai–Tibet Plateau, hyperuricaemia, prevalence rate, high-altitude polycythaemia, altitude exposure time, altitude exposure form

## Abstract

**Background:**

Hyperuricaemia is associated with increased metabolic syndrome, cardiovascular disease, and mortality risk in the adult population and is more common in plateau areas. Han male immigrants are a high-risk population and deserve more attention. The correlations between different altitude exposure durations and exposure modalities with hyperuricaemia have not yet been reported.

**Methods:**

In this cross-sectional study, qualified subjects were selected from two units on the Qinghai–Tibet Plateau and underwent a questionnaire survey (age, altitude exposure time, altitude exposure form), anthropometric measurements (body mass index [BMI], blood pressure [BP], and heart rate [HR]), and laboratory tests (white blood cell count [WBC], haemoglobin [HB], platelet count [PLT], and serum uric acid [SUA]). Univariate and multivariate logistic regression models were used to detect factors associated with hyperuricaemia.

**Results:**

The overall prevalence of hyperuricaemia was 40.8% (73 cases) among 179 Han male immigrants. Multivariate logistic regression analysis revealed that hyperuricaemia was significantly related to altitude exposure time (OR 6.744, 95% CI 1.263–36.019), altitude exposure form (OR 2.580, 95% CI 1.068–6.231) and high-altitude polycythaemia (OR 2.125, 95% CI 1.011–4.465).

**Conclusion:**

High-altitude polycythaemia, exposure to high altitudes for 1–5 years, and long-term exposure to the same high-altitude areas when the high-altitude exposure dose is equal are important factors associated with hyperuricemia.

## 1. Introduction

Hyperuricaemia is a metabolic disease that is not only the main cause of gout [1] but also increases the risk of developing hypertension, obesity, hyperlipidaemia, cardio-cerebral vascular diseases and chronic kidney disease [2–7]. The prevalence of hyperuricaemia varies worldwide due to differences in environmental, lifestyle and genetic factors. High-altitude hypoxia disrupts purine metabolism, leading to increased uric acid production. Concurrently, it inhibits Na^+^-K^+^-ATPase activity in renal tubular epithelial cells and induces inflammatory responses and apoptosis. These collectively cause secondary renal injury and compromise uric acid excretion, and consequently resulting in significantly elevated serum uric acid levels [8–10]. The prevalence of hyperuricaemia among local residents living on the Qinghai–Tibet Plateau for a long period has been as high as 40.7% [4]. The prevalence of hyperuricaemia in the Han population, who migrate from plains to high-altitude areas, is greater than that in Tibetan residents, which may be related to the different tolerances and adaptabilities to hypoxia caused by genetic differences [11].

The level of serum uric acid is influenced by many factors, including age, sex, race, eating habits, BMI and the environment [12–16]. Previous studies have reported the prevalence of and risk factors for hyperuricaemia among immigrants on the Qinghai–Tibet Plateau. The prevalence of hyperuricaemia in men is significantly greater than that in women (59.9% for men and 22.5% for women). The age group with the highest prevalence of hyperuricaemia in men is 21-25 years. In addition to traditional risk factors such as age and sex, polycythaemia is also a risk factor for hyperuricaemia in this group [11]. Research has also reported that in Ganzi Tibetan Autonomous Prefecture, Sichuan Province, for men, low education level, current drinking, high BMI and high serum creatinine are risk factors for hyperuricaemia. Participating in regular, high levels of physical activity and being in the Tibetan ethnicity group (rather than the Han ethnicity group) were protective factors against hyperuricaemia [17].

Due to the effects of high-altitude hypoxia, hyperuricaemia occurring on the plateau leads to more severe kidney damage and cardiovascular/cerebrovascular diseases compared to lowland areas, often exacerbating concurrent conditions such as hypertension and high-altitude heart disease [4,18]. The Han population has become a key contributor to plateau development. Existing reports indicate that the prevalence of hyperuricaemia among Han people on the plateau is higher than that among Tibetans. Beyond genetic background, studies have identified sex, age, and high-altitude polycythaemia as risk factors for hyperuricaemia in the Han population on the plateau. Among employees on the Qinghai-Tibet Plateau, some commute between different altitudes, whereas others are stationed long-term at a fixed altitude. Additionally, their total duration of high-altitude exposure varies. However, previous research has not focused on the correlations between the duration and modality of high-altitude exposure with hyperuricaemia. This warrants thorough investigation to develop more targeted health guidance programs.

## 2. Subjects and methods

### 2.1. Study design and research subjects

A cross-sectional study was conducted from February 2024 to May 2024 involving representative Han male immigrants selected from two units on the Qinghai–Tibet Plateau. The participants were 18–50 years old and had been exposed to the plateau for at least one month. The participants were people who resided at 3800 metres and frequently commuted between 2800 and 4200 metres (working at 4200 metres from Monday to Friday and returning to 2800 metres for rest on Saturday and Sunday). All participants exclusively consumed meals prepared by the institutional canteen during the study period. To standardize purine intake, a 3-day low-purine dietary intervention was implemented prior to venous blood collection. This protocol included the following rigorous measures: canteen chefs were provided with specialized low-purine menus developed by clinical nutritionists who prohibited high-purine ingredients, and standardized meals were supplied to all participants. All of whom performed equal amounts of exercise. The exclusion criteria were as follows: (1) patients with incomplete clinical data; (2) patients with diabetes, hyperlipidaemia or cardiac or renal insufficiency; and (3) patients who were taking drugs that affect serum uric acid levels before they were included in the study. According to the inclusion and exclusion criteria, 179 people were ultimately enrolled, and the basic demographic information and laboratory examination results of the subjects were collected. This study was conducted according to the guidelines of the Declaration of Helsinki and approved by the Ethics Committee of the General Hospital of Western Theater command (2024EC6-ky006), and all of the patients provided written informed consent.

### 2.2. Data collection and anthropometry

Information on age, altitude exposure time, altitude exposure form, and physical activity level was collected *via* self-administered questionnaires during individual interviews. Trained medical professionals measured height, weight, and blood pressure (BP). Body mass index (BMI) was calculated as weight in kilograms divided by height in meters squared (kg/m^2^). Prior to BP measurement, the participants were seated and rested for at least five minutes. Systolic blood pressure (SBP) and diastolic blood pressure (DBP) were then measured in the seated position using an automatic sphygmomanometer, with the average of three consecutive readings recorded.

### 2.3. Laboratory measurements

After fasting for at least 8 h, venous blood samples were collected, and white blood cell counts, red blood cell counts, haemoglobin counts, platelet counts, and serum uric acid levels were measured.

### 2.4. Definitions

HAPC was defined as an Hb concentration ≥ 210.0 g/L (males) [19]; hyperuricaemia was defined as a blood uric acid level ≥ 420.0 μmol/L (males) [20]; and hypertension status was defined as an SBP ≥ 140 mmHg and/or a DBP ≥ 90 mmHg measured three times on different days without the use of antihypertensive drugs [21]. High altitude is considered an altitude greater than 2500 m above sea level [22].

### 2.5. Statistical methods

The categorical variables are presented as cases (n) and percentages (%), the normally distributed measurement variables are presented as the means ± SDs, and the nonnormally distributed measurement variables are presented as medians (P25, P75). Comparisons between two groups were performed with the Pearson χ^2^ test or Fisher’s exact test for categorical variables, Student’s t test for normally distributed variables, and the Mann–Whitney U test for nonnormally distributed variables. The weekly high-altitude exposure doses for the two different forms of exposure are presented as areas under the curve (AUC) [23]. Based on GPS-recorded altitude data, the integral under the weekly altitude-time curve (unit: m·d) was calculated using the trapezoidal rule, representing the integrated effect of altitude intensity and exposure duration. Binary logistic regression analysis was used to identify independent risk factors for hyperuricaemia among parameters that differed between the two groups in the univariate analysis. Odds ratios (ORs) and 95% confidence intervals (CIs) were calculated. IBM SPSS statistics 26.0 was used for statistical analysis, and *p* < 0.05 was considered to indicate statistical significance.

## 3. Results

### 3.1. Baseline characteristics and univariate analysis

A total of 179 Han male employees on the Qinghai–Tibet Plateau were included in this study, and the prevalence of hyperuricaemia was 40.8% (73 cases). Univariate analysis revealed that hyperuricemia status was significantly associated with HAPC status, altitude exposure time, altitude exposure form and white blood cell count (Table 1).

**Table 1.** Comparison of indicators between the hyperuricaemia group and Non-Hyperuricaemia group.

		All	Non-hyperuricemia	Hyperuricemia	*χ^2^/t/z*	*P* value
All		179	106(59.20)	73(40.80)		
Age (years), n(%)	≤20	28(15.64)	12(42.86)	16(57.14)	77.229^a^	0.065
	>20 and ≤30	73(40.78)	40(54.79)	33(45.21)		
	>30 and ≤40	55(30.73)	37(67.27)	18(32.73)		
	>40	23(12.85)	17(73.91)	6(26.09)		
Exposure time (years),n(%)	<1	13(7.30)	11(84.62)	2(15.38)	14.292^a^	0.003
	≥1 and ≤5	82(45.80)	37(45.12)	45(54.88)		
	>5 and ≤10	59(33.00)	39(66.10)	20(33.90)		
	>10	25(14.00)	19(76.00)	6(24.00)		
Exposed form, n(%)	resident	73(40.80)	32(43.84)	41(56.16)	12.078^a^	0.001
	frequent commuting	106(59.20)	74(69.81)	32(30.19)		
Hypertension, n(%)	No	98(54.75)	55(56.12)	43(43.88)	0.859^a^	0.354
	Yes	81(45.25)	51(62.96)	30(37.04)		
HR (times/min)	median(P_25_,P_75_)	80.00(72.00,88.00)	80.0(72.75,89.25)	80.0(71.50,87.00)	−0.711^c^	0.477
HAPC	No	133(74.30)	85(63.91)	48(36.09)	4.718^a^	0.030
	Yes	46(25.70)	21(45.65)	25(54.35)		
PLT (×10^9^/L)	median(P_25_,P_75_)	212.00(177.00,254.00)	215.50(177.75,261.25)	198.00(176.00,241.50)	−1.548^c^	0.122
WBC (×10^9^/L)	median(P_25_,P_75_)	8.20(7.00,9.40)	7.90(6.80,9.23)	8.30(7.50,9.70)	−1.983^c^	0.047
BMI (kg/m^2^), n(%)	<24	110(61.45)	67(60.91)	43(39.09)	1.174^a^	0.556
	≥24 and <28	52(29.05)	31(59.62)	21(40.38)		
	≥28	17(9.50)	8(47.06)	9(52.94)		

HR: Heart rate, HAPC: high-altitude polycythemia, RBC: Red blood cell, PLT: Blood platelet, WBC: White blood cell, a: x^2^ test, b: 2-sample t test, c: Mann–Whitney U test, d: Fisher exact test.

### 3.2. Area under the curve of different altitude exposure forms

**Figure 1.**
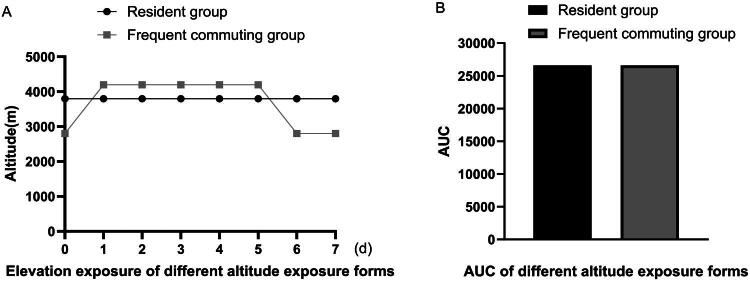
AUC of weekly altitude exposure dose for different forms. Different altitude exposure forms include those who reside at 3800 metres(resident group)and those who frequently commute between 2800 and 4200 metres(frequent commuting group). The weekly altitude-time curve (unit: m·d) was calculated using the trapezoidal rule, representing the integrated effect of altitude intensity and exposure duration. A: the daily exposure altitude within a week in two groups. B: the AUC of the weekly altitude exposure dose for two groups. AUC: area under the curve.

Different forms of high-altitude exposure include those who reside at 3800 metres and those who frequently commute between 2800 and 4200 metres. The area under the curve of the weekly high-altitude exposure dose for the two different forms of exposure did not significantly differ (Figure 1).

### 3.3. Multivariate analysis

The multivariate logistic regression analysis adjusted for factors such as age and BMI revealed that exposure to high altitudes for 1–5 years (OR = 6.744, 95% CI 1.263–36.019), HAPC status (OR = 2.125, 95% CI 1.011–4.465) and long-term exposure to the same high-altitude areas (OR = 2.580, 95% CI 1.068–6.231) when the exposure dose was equal at high altitudes were important factors associated with the development of hyperuricaemia in Han male immigrants in the Qinghai–Tibet Plateau region (Table 2).

**Table 2.** Multivariate regression analysis of factors influencing hyperuricaemia.

	OR	95%CI	*P* value
Altitude exposure time (years), n(%)			
<1	1		
≥1 and ≤5	6.744	1.263-36.019	0.026
>5 and ≤10	3.405	0.622-18.641	0.158
>10	2.305	0.348-15.282	0.387
HAPC (Yes)	2.125	1.011-4.465	0.047
Altitude exposure form, n(%)			
Frequent commuting	1		
Resident	2.580	1.068-6.231	0.035
Age (years), n(%)			
≤20	1		
>20 and ≤30	1.130	0.415-3.079	0.811
>30 and ≤40	1.519	0.402-5.742	0.538
>40	0.858	0.182-4.057	0.847
BMI (kg/m^2^), n(%)			
<24	1		
24-28	1.258	0.583-2.715	0.559
≥28	2.275	0.717-7.217	0.163
WBC (×10^9^/L)	1.186	0.996-1.413	0.056

OR: Odds ratio. HAPC: high-altitude polycythemia.

### 3.4. Serum uric acid levels and hyperuricaemia prevalence rates of han male immigrants with different durations of altitude exposure

**Figure 2.**
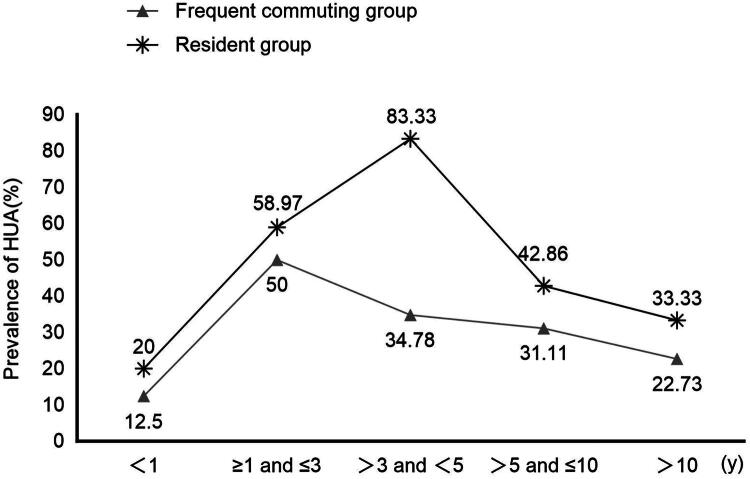
Hyperuricaemia prevalence with different altitude exposure time. Resident group: Han male immigrants who reside at 3800 metres. Frequent commuting group: Han male immigrants who frequently commute between 2800 and 4200 metres. Prevalence of HUA (%): prevalence of hyperuricaemia.

The serum uric acid level and prevalence of hyperuricaemia in different altitude exposure groups during different periods of high-altitude exposure are shown in Table 3 and Figure 2. The prevalence rate and serum uric acid level of both groups were the highest after exposure to the plateau for 1–5 years. The prevalence rate of hyperuricaemia among people who frequently commuted between 2,800 metres and 4,200 metres peaked (50%) at ≥1 year and ≤3 years, and the prevalence rate of hyperuricaemia among people who resided at 3800 metres peaked (83.33%) at > 3 years and ≤5 years.

**Table 3.** Serum uric acid level and prevalence of hyperuricaemia.

	Frequent commuting			Resident		
Exposure time	N	SUA( $\bar{x}$ ±s)	HUA%(95%CI)	N	SUA( $\bar{x}$ ±s)	HUA%(95%CI)
<1	8	322.68 ± 86.53	12.5(0,35.42)	5	404.46 ± 54.52	20(0,55.06)
≥1 and ≤5	31	402.74 ± 88.62	38.71(21.56,55.86)	51	442.83 ± 72.29	64.71(51.59,77.82)
≥1 and ≤3	8	400.75 ± 104.91	50(15.35,84.65)	39	440.46 ± 75.80	58.97(43.54,74.41)
>3 and ≤5	21	403.43 ± 84.89	34.78(15.32,54.25)	12	450.56 ± 61.77	83.33(62.25,100.00)
>5 and ≤10	45	376.97 ± 71.98	31.11(17.58,44.64)	14	423.16 ± 75.34	42.86(16.93,68.78)
>10	22	375.80 ± 90.85	22.73(5.22,40.24)	3	376.71 ± 60.17	33.33(0,86.68)

SUA: Serum uric acid, HUA%: Prevalence of hyperuricemia.

## 4. Discussion

In this study, we investigated the prevalence of hyperuricaemia in 179 Han male immigrants on the Qinghai–Tibet Plateau and analysed the factors associated with hyperuricaemia. The overall prevalence rate of hyperuricaemia in this group was 40.8% (73 patients), and it was highest among people aged ≤20 years (57.14%); moreover, prevalence decreased with age. The prevalence rate of hyperuricaemia was the highest among people exposed to high altitudes for 1–5 years (54.88%), and after more than 5 years, the prevalence rate gradually decreased with increasing altitude exposure time. Multivariate logistic regression analysis revealed that the factors associated with hyperuricaemia in this group included altitude exposure time, altitude exposure form and HAPC status.

This study revealed that hyperuricaemia in Han male immigrants on the Qinghai-Tibet Plateau was associated with the duration of altitude exposure. The prevalence of hyperuricaemia was the highest among people exposed to high altitudes for 1–5 years, and the association was 6.744 times greater than that of people exposed to high altitudes for less than 1 year. The association with hyperuricaemia gradually decreased in the population exposed to high altitudes for more than 5 years but less than or equal to 10 years and for those exposed to high altitudes for more than 10 years, with no significant difference compared with the population exposed to high altitudes for less than 1 year. The trend of the change in the hyperuricaemia prevalence rate with increasing altitude exposure time in this population may be related to the physiological adaptation of the body to high-altitude environments. Hypoxia at high altitudes can affect serum uric acid levels in many ways, such as increasing xanthine oxidase activity mediated by hypoxia-inducible factor (HIF-1α), which elevates purine metabolites, and the induction of secondary kidney injury through oxidative stress and apoptosis, which reduces uric acid excretion [8,9,24]. For individuals in the Han population, the body starts a series of stress compensation reactions, such as a significant increase in the P50 value to maintain the normal tissue oxygen level, in the early stage (i.e. within 3–7 days after entering the plateau). After adapting to the plateau environment for 30 days, the P50 value gradually decreases [9]. Some studies have also suggested that the degree and duration of exposure to high altitude affect the glomerular filtration rate, renal blood flow and filtration fraction, thus affecting the degree and duration of renal physiological adaptation [25]. Therefore, the mechanisms of high-altitude physiological adaptation are multifactorial. Currently, no relevant literature has reported the specific adaptation time for uric acid metabolism. This study found the highest hyperuricemia prevalence at 1–5 years of high-altitude exposure, laying the groundwork for time-stratified management strategies.

The group we observed included people who reside at 3800 metres and people who frequently travelled between 2800 and 4200 metres. To accurately assess the high-altitude exposure dose, the weekly cumulative altitude exposure dose was quantified using the area under the curve (AUC). Based on GPS-recorded altitude data, the integral under the weekly altitude-time curve (unit: m·d) was calculated using the trapezoidal rule, which represents the integrated effect of altitude intensity and exposure duration [23]. The area under the curve of the weekly high-altitude exposure dose for the two different forms of exposure was equal. This study revealed that in this group, staying at 3800 metres was an important factor associated with hyperuricaemia, and for those individuals, the risk of developing hyperuricaemia was 2.580 times greater than that of people who frequently travel between 2800 and 4200 metres. We further analysed the changes in the prevalence of hyperuricaemia and serum uric acid with increasing exposure time at high altitudes in the two groups. The results revealed that both groups had the highest serum uric acid level and the highest prevalence rate of hyperuricaemia during the exposure time of 1–5 years at high altitude. Further analysis revealed that the prevalence of hyperuricaemia in the frequent commuting group reached its peak at 1-3 years, whereas that in the residential group reached its peak at 3-5 years. However, the prevalence of hyperuricaemia in the residential group was greater than that in the frequent commuting group at both 1-3 years and 3-5 years. Previous studies have demonstrated that hypoxic preconditioning reinforces HIF-alpha-dependent HSP70 signalling to reduce ischemic renal failure-induced renal tubular apoptosis and autophagy [26]. There have also been reports indicating that intermittent hypoxia conditioning fosters beneficial physiological responses across multiple organs and systems [27]. It is postulated that cyclic variations in hypoxic stimulation during cross-altitude exposure accelerate HIF pathway responsiveness, enabling the organism to acquire adaptive mechanisms more rapidly, whereas sustained exposure to a fixed altitude delays adaptation due to persistent chronic hypoxia. This discovery offers novel perspectives for the prevention and management of hyperuricaemia in high-altitude regions through a dual temporal-spatial framework of intervention.

Due to limitations in the study conditions, this research did not observe objective indicators such as partial pressure of oxygen, oxygen saturation, pulmonary function, or cardiac function, and thus lacks objective evidence to reflect the body’s adaptability. Therefore, our hypothesis regarding physiological adaptation is merely speculative. We hope to improve the testing items in subsequent studies to contribute to exploring the mechanisms underlying the susceptibility to hyperuricaemia among the Han male population in high-altitude areas.

Long-term exposure to an anoxic environment leads to a compensatory increase in red blood cells. HAPC is a common form of chronic mountain sickness (CMS) in high-altitude populations that can lead to secondary physical injuries such as thromboembolism and kidney injury [25,28]. Previous studies have shown that the prevalence of hyperuricaemia and increased serum uric acid levels at high altitudes are closely related to haemoglobin levels and polycythaemia status. This study also revealed that HAPC status (Hb ≥ 210 g/L) is an important factor associated with the development of hyperuricaemia among Han male immigrants on the Qinghai–Tibet Plateau. High-altitude polycythaemia may aggravate tissue hypoxia and secondary haemodynamic changes, which are conducive to increasing nucleic acid synthesis and uric acid production, as well as the occurrence of secondary renal injury [21,28], thus leading to hyperuricaemia.

This study revealed that the prevalence of hyperuricaemia among Han male immigrants on the Qinghai–Tibet Plateau decreased gradually with increasing age, which was consistent with the findings of previous studies on male populations on plateaus [12,29]. However, in the present study, we did not detect a significant correlation between age and the prevalence of hyperuricaemia, which may be related to the small sample size. It is also possible that the older the population is, the longer the exposure time at high altitude, which leads to the adaptation of the body to the environment, reducing the influence of age and increasing the influence of exposure time at high altitude.

There are several limitations in this study. First, it was a cross-sectional study, and causality could not be assessed. Second, the sample size was small, there may have been sampling errors, and this study restricted the participants to Han males aged 18–50 years, which limits the generalizability of the findings to broader populations (including females, other age groups, and different ethnic groups). Third, the previous levels of uric acid among participants were not collected; furthermore, the effects of previous dietary habits, renal function, body composition and familial-related diseases on hyperuricaemia were not included in this study. Thus, multicentre and prospective clinical studies with larger samples are needed to verify these findings and explore the underlying mechanisms.

## 5. Conclusion

In summary, this cross-sectional study of 179 Han male immigrants on the Qinghai–Tibet Plateau revealed a high prevalence rate of hyperuricaemia among this group, and altitude exposure for 1–5 years, HAPC status, and long-term exposure to the same high-altitude areas when the exposure dose is equal at high altitudes are important factors associated with the development of hyperuricaemia. Hyperuricaemia has become an important public health issue for Han male immigrants in the Qinghai–Tibet Plateau region. It is necessary to establish better prevention and treatment strategies for hyperuricaemia. In the process of adapting to high-altitude environments, the exposure time and form at high altitudes are particularly important, and the potential underlying mechanism is still poorly understood. The results of this study may be helpful for designing further research on altitude adaptation and improving medical care strategies for high-altitude populations.

## Data Availability

Data are available on request by the corresponding author (Yue Cheng) due to privacy/ethical restrictions.
